# Multiple primary lung cancer: Updates of clinical management and genomic features

**DOI:** 10.3389/fonc.2023.1034752

**Published:** 2023-02-22

**Authors:** He Tian, Guangyu Bai, Zhenlin Yang, Ping Chen, Jiachen Xu, Tiejun Liu, Tao Fan, Bingning Wang, Chu Xiao, Chunxiang Li, Shugeng Gao, Jie He

**Affiliations:** ^1^ Department of Thoracic Surgery, National Cancer Center/National Clinical Research Center for Cancer/Cancer Hospital, Chinese Academy of Medical Sciences, and Peking Union Medical College, Beijing, China; ^2^ Department of Medical Oncology, Yancheng No. 1 People’s Hospital, Yancheng, Jiangsu, China; ^3^ Department of Medical Oncology, National Cancer Center/National Clinical Research Center for Cancer/Cancer Hospital, Chinese Academy of Medical Sciences, and Peking Union Medical College, Beijing, China; ^4^ Department of Pathology, National Cancer Center/National Clinical Research Center for Cancer/Cancer Hospital, Chinese Academy of Medical Sciences, and Peking Union Medical College, Beijing, China

**Keywords:** multiple primary lung cancer (MPLC), clinical management, genomic, heterogeneity, intrapulmonary metastasis (IPM)

## Abstract

In recent decades, multiple primary lung cancer (MPLC) has been increasingly prevalent in clinical practice. However, many details about MPLC have not been completely settled, such as understanding the driving force, clinical management, pathological mechanisms, and genomic architectures of this disease. From the perspective of diagnosis and treatment, distinguishing MPLC from lung cancer intrapulmonary metastasis (IPM) has been a clinical hotpot for years. Besides, compared to patients with single lung lesion, the treatment for MPLC patients is more individualized, and non-operative therapies, such as ablation and stereotactic ablative radiotherapy (SABR), are prevailing. The emergence of next-generation sequencing has fueled a wave of research about the molecular features of MPLC and advanced the NCCN guidelines. In this review, we generalized the latest updates on MPLC from definition, etiology and epidemiology, clinical management, and genomic updates. We summarized the different perspectives and aimed to offer novel insights into the management of MPLC.

## Introduction

Lung cancer is the most fatal malignancy globally, significantly burdening the public health system (1). In recent decades, lung cancer risk has increased along with the prolonged lifespan of people (2). Meanwhile, the prevalence of low-dose computed tomography (LDCT) lung cancer screening programs boosted lung cancer diagnosis (3).

Multiple primary lung cancer (MPLC), indicating patients with multiple tumor sites of independent origins in the lung, which can be synchronous or metachronous (4), is becoming a common phenomenon in clinical practice. In 1975, Martini and Melamed outlined the first clinical and pathological criteria for MPLC (M-M criteria) based on 108 cases (5). Based on the M-M criterion, many researchers have put much effort into this kind of disease.

The definite etiology and epidemiology of this disease remain unclear, although previous studies have reported that smoking (6), field cancerization (7–9), and familial heredity (10) were factors tightly associated with the initiation of MPLC. Moreover, choosing the best therapy, studying genomic mutations and architectures (11), distinguishing intrapulmonary metastasis (IPM) from MPLC (12), and understanding the tumor microenvironment of MPLC (13) are still research hotspots.

What we know about the MPLC is far from enough. From the perspective of diagnosis and clinical management, distinguishing MPLC from IPM is vital and may seriously affect the treatment strategy. Nowadays, preoperative imaging evidence can no longer accurately distinguish these two diseases. Therefore, distinguishing MPLC from IPM always expects genomic technology (14–16). Besides, surgical resection is still the mainstream treatment choice for MPLC patients. Researchers spent years searching for the best surgical time and resection strategy for patients (17). However, for patients who are not suitable for surgery, non-operative therapy such as image-guided thermal ablation (IGAB) and stereotactic ablative radiotherapy (SABR) were considered (18–20). And in recent years, immunotherapy for multiple GGOs (ground glass opacities) attracted much attention, while the curative effect is not ideal (21, 22). From the perspective of genomic profiling, several previous researchers have reported the enormous genetic heterogeneity among the multiple lesions of MPLC using next-generation sequencing (11, 15, 23). Moreover, exploring the origin and initiation mechanisms of MPLC will also need the help of genomic profiling or other technologies such as RNA sequencing and single-cell sequencing (24).

In this review, we will give a comprehensive scenario of MPLC. We tried to generalize the latest updates on MPLC from definition, etiology and epidemiology, clinical management, and genomic updates. And we hope our work can open novel avenues for the major concerns of MPLC and advance the management of this unsolved disease.

## Definition of MPLC

The definition and staging process of MPLC started with Martini and Melamed. They outlined the first clinical and pathological criteria for MPLC (M-M criteria) (5). In the M-M criteria, for synchronous MPLC, tumors should be physically separate, histologically identical, or different. If the tumors were the same histologically, they should originate from carcinoma *in situ* or without metastases. For metachronous MPLC, the tumors could be histologically heterogeneous or the same. If they were of the same histology, the intervals should be at least two years, or the tumors were physically distinct and without metastases, or they should originate from carcinoma *in situ*. The M-M criteria were used for 20 years until the revised definition of MPLC was brought up by Antakli et al. (25) in 1995. The underlying logic of the M-M criteria and Antakli’s standard were similar, while Antakli was more concise. In 2016, the International Association for the Study of Lung Cancer (IASLC) classified multifocal lung nodules into four types based on histology judgment, clinical information (including lesion location and radiologic features), and prognosis (26): *Type I*-Second primary lung cancers, *Type II*-Separate tumor nodules (intrapulmonary metastasis), *Type III*-Multifocal lung adenocarcinoma with ground glass/lepidic features, and *Type IV*-Pneumonic-type lung adenocarcinoma. Most clinicians classified second primary lung cancers (*Type I*) and Multifocal lung adenocarcinoma with ground glass/lepidic feature (*Type III*) into MPLC. In second primary lung cancers (*Type I*), each tumor is of independent biological origin. Thus, in clinical practice, separate T, N, and M stage should be determined for each lesion, like individual tumors. For multifocal lung adenocarcinoma with ground glass/lepidic feature (*Type III*), which belongs to ground-glass opacity (GGO), IASLC suggested assigning the T stage according to the highest T lesions and the N and M stages for all lesions collectively, and we summarized the criteria identifying them in **Table 1**. So far, the current classification of MPLC from IASLC has been widely accepted, offering much support for establishing the diagnosis criteria for MPLC. In 2022, the NCCN guidelines (NSCLC Version 3.2022) emphasized that the MPLC diagnosis requires a multidisciplinary setting involving surgeons, pathologists, radiation oncologists, and medical oncologists. In addition, they suggested some approaches to distinguish MPLC and IPM, which will be discussed later in this review.

**Table 1:** Multifocal ground glass/lepidic lung adenocarcinoma diagnosis criteria according to the 8th edition of TNM classification.

	Definition of multifocal GG/L LUAD	Application
**Clinical Criteria**	There are multiple subsolid nodules (either pure ground glass or part solid), with at least one suspected (or proved) to be cancer.	This applies whether or not a biopsy has been performed on the nodules.
		This applies if the other nodules(s) are found by biopsy to be AIS, MIA, or LPA.
		This applies if a nodule has become >50% solid but is judged to have arisen from a GGN, provided that there are other subsolid nodules.
		GGN lesions <5 mm or lesions suspected to be AAH are not counted.
**Pathologic Criteria**	There are multiple foci of LPA, MIA, or AIS.	This applies whether a detailed histologic assessment (i.e., proportion of subtypes, etc.) shows a matching or different appearance.
		This applies if one lesion(s) is LPA, MIA, or AIS and there are other subsolid nodules of which a biopsy has not been performed.
		This applies whether the nodule(s) are identified preoperatively or only on pathologic examination.
		Foci of AAH are not counted.

A radiographically solid appearance and the specific histologic subtype of solid of adenocarcinoma denote different things.

AAH, atypical adenomatous hyperplasia; AIS, adenocarcinoma in situ; GGN, ground glass nodule; LPA, lepidic-predominant adenocarcinoma; MIA, minimally invasive adenocarcinoma.

## Distinguishing MPLC from IPM

In the process of defining MPLC, IPM diagnosis is often accompanied. Although the M-M criteria involve pathological diagnosis, it is still important to distinguish between MPLC and IPM before treatment. If MPLC is wrongly diagnosed as IPM, the patient may lose the opportunity for surgery or receive chemical and radiation damage caused by unnecessary doses (27). Conversely, if IPM is diagnosed as MPLC, the patient’s survival will be affected (11). Previously, the differential diagnosis of MPLC and IPM was mainly based on histopathological evidence. However, the accuracy of pathological diagnosis is limited, and the accuracy of the M-M standard has been proven to be relatively low (28). The pathological diagnosis standard proposed by Girard et al. in 2009 is considered as accurate as 91% and received wide acceptance (28, 29). Since the diffusion of next-generation sequencing, the diagnosis at the gene level is indispensable to distinguishing the two diseases. We will elaborate these topics more specifically in the following article.

## Etiology and epidemiology

The hypotheses of MPLC etiology are various. Tobacco use (including passive smoking by never-smokers) (6) and familial inheritance (9, 10) are the two main points. Field cancerization was also reported to be a likely mechanism of multifocal lung cancer (30, 31). In addition, a previous study reported that lung cancer survivors were more vulnerable to subsequent pulmonary malignancies (32). In 2021, Chen et al. first demonstrated that microplastic exposure was associated with the etiology of multiple pulmonary GGOs (ground-glass nodules) (33), linking MPLC pathogenesis to environmental factors. Genetic variants, such as EGFR germline mutations, were also reported to be the driving force of MPLC (15, 34). A SEER (Surveillance Epidemiology and End Results) analysis suggested that younger age, female gender, earlier stage, and white race are risk factors for MPLC (35). Previous studies reported that the incidence of multi-focal lung cancer ranges from 0.2% to 20% (36–38). We believe that the incidence of MPLC in the real world is far more than what has been reported. Since the COVID-19 (Corona Virus Disease-19) pandemic in 2019, the number of patients undergoing pulmonary CT (computerized tomography) increased, resulting in a wave of the rapid growth of MPLC (39). So far, the associations between COVID-19 and MPLC have not been illustrated.

## Clinical management and prognosis

Nowadays, there are multiple treatments for MPLC, including surgery, stereotactic ablative radiation (SABR), immunotherapy, and ablation. If the multiple lung tumors were diagnosed as IPM, the treatment strategy should obey the principle of treating T3/T4 NSCLC. In this part, we collected the relevant studies on MPLC and compared the efficacies and limitations of different treatments, which are summarized in **Table 2**.

**Table 2:** Summary of MPLC clinical managements.

Management	Supporting studies (PMID)	Efficacies	Limitations
**Surgery**	31032071, 25725930, 21145616, 31376378, 31795997, 26602007, 27293837, 33243621, 30345106	Surgical resection remains the mainstream treatment for MPLC. Lobectomy and sublobectomy are both acceptable. Pneumonectomy should be avoided.	There is still a lack of prospective randomized controlled trials based on large samples.
**SABR**	23746675, 30477740, 33645424	SABR is safe and acceptable in the treatment of multiple primary lung cancer, and the efficacies of SABR in MPLC and single lung cancer are with no statistical difference in survival, recurrence and local failure rate.	There are few studies focusing on SABR in MPLC and more evidence is warranted.
**Ablation**	31402333, 22413004, 33152627	IGTA technique is an effective and safe technique for the treatment of MPLC.	The evidence level of IGTA in MPLC treatment is low.

PMID, PubMed Unique Identifier. SABR, stereotactic body radiationtherapy; IGTA, image-guided thermal ablation; MPLC, multiple primary lung cancer.

For surgery, previous researchers have devoted much effort to identifying the most appropriate resection strategy and risk factors of prognosis for MPLC (40). Both synchronous and metachronous MPLC can benefit from surgical resection (41–43). In 2019, Chen et al. suggested that sub-lobar resection can lead to an equivalent prognosis to standard resection (lobectomy) and was beneficial in preserving pulmonary function (44), and for both ipsilateral and bilateral MPLC, surgery resection is as safe as single-nodule lung cancer (43, 45). It is believed that radical pneumonectomy should be avoided, if possible, for it may lead to a poor prognosis (46, 47). It’s worth mentioning that the situation becomes more complicated when GGO/lepidic lesions coexist with solid or subsolid nodules. In clinical practice, removing solid nodules might be the best choice and at the same time, removing the other GGOs on the same side is more helpful for diagnosing and staging (48).

For patients with MPLC, surgery is far from a single treatment. On the one hand, there are many high-risk patients of older age or with underlying cardiopulmonary comorbidities, for whom thoracic operations might lead to severe intraoperative and postoperative complications. On the other hand, some MPLC patients have lung nodules disseminated in more than one lobe, especially for multifocal lung adenocarcinoma with ground glass/lepidic features (Type III), which are inert in nature compared to solid and subsolid nodules. Removing all nodules seems impossible for these people or will lead to much lung function loss. Besides, the selection logic of resection strategy is different among different surgeons, for removing tumors to the maximum extent is as essential as guaranteeing patients’ post-treatment life quality.

For MPLC patients who are not suitable for surgery, stereotactic ablative radiation (SABR), and image-guided thermal ablation (IGTA) (18–20, 49) are recommended. The ESMO guidelines for early and locally advanced NSCLC recommended that MPLC should be mainly assessed with curative intent (complete resection), while SABR was also an effective choice (50). Previous research showed that stereotactic ablative body radiotherapy achieved an 84% lesion control rate after two years in MPLC (19). And Nikittas et al. compared the effectiveness and safety between MPLC patients and single nodule patients. They suggested improved disease and survival outcomes in patients receiving both surgery and SBRT and multiple courses of SBRT alone for MPLC, though this could be due to selection bias (18). Image-guided thermal ablation is a method that has been proven to be applicable to the treatment of pulmonary nodules in recent years (20, 51). And it contains radiofrequency ablation (RFA), microwave ablation (MWA), and cryoablation (CA) (51). Aufranc et al. (52) studied 115 patients with a total of 160 lung tumors to compare microwave (MWA) and radiofrequency (RFA) ablation in the percutaneous treatment of primary and secondary lung tumors. They found that MWA and RFA are effective and safe IGTA techniques for the treatment of lung cancer. Besides, there are other retrospective studies that improved the safety and efficiency of IGTA (53–55), but they are small-sized cohort studies.

Many factors are associated with better survival in MPLC, including small tumor size (size of the largest tumor and sum of tumor sizes, also presenting as SUVmax value in radiologic images) (56, 57), similar histology of multiple lesions, N0 stage (better prognosis) (58), T1 stage (42), female gender (59), young age, non-smoker and FEV1/FVC ≥70% (42). Evidence showed that male gender, symptomatic disease, and lymph node involvement were independent factors for adverse prognosis (60). The prognosis of stage I synchronous multiple primary non-small cell lung cancer is similar to that of solitary primary NSCLC (61). To take precautions against metachronous primary lung cancers, regular CT examination after surgical resection is recommended for heavy smokers (62). Long-term follow-up for both synchronous and metachronous MPLC is necessary by which potentially curable secondary lung cancers can be identified when they are still under control (63).

Taken together, although people spent years finding the best treatment for MPLC, surgical resection remains the mainstream, while the non-surgical treatment approaches for MPLC are also indispensable.

## Clinical trials about MPLC

To the best of our knowledge, nine registered clinical trials about MPLC have been launched so far (**Table 3**), half of which are interventional, and the other half are observational. Most clinical trials are still recruiting patients, and no trial has been completed. The combination of immunotherapy and ablation-related methods are under the spotlight. The newest trial was launched on 22^nd^ December 2022, by Li et al. from Ruijin Hospital, Affiliated to Shanghai Jiao Tong University School of Medicine. They aim to compare ablation plus surgery with surgery alone in treating bilateral MPLC. Jie et al. from the First Affiliated Hospital of Xiamen University, China, launched clinical trials of inoperable synchronous MPLC, applying stereotactic ablation radiotherapy and Sintilimab. At the same time as Jie et al., Chen et al. from Shanghai Pulmonary Hospital, China, started a phase II clinical trial of MPLC using microwave ablation plus Camrelizumab. To evaluate the diagnostic value of 18F-FDG PET/CT dynamic imaging of distinguishing MPLC from IPM, Jin et al. from Fifth Affiliated Hospital, Sun Yat-Sen University, started a clinical trial including 120 participants in 2018, which is estimated to be completed in 2024.

**Table 3:** Registered clinical trials about MPLC.

Trials	Alias	Study Type	Phase	Participants	Estimated Enrollment	Interventions	Primary Outcome	Status	Principal Investigator
NCT04840758		Interventional	I/II	Early Inoperable sMPLC	39	SABR plus Sintilimab	Abscopal effect rate	Not yet recruiting	Jiang jie, MD First affiliated Hospital of Xiamen University
NCT05053802	MAGIC	Interventional	II	MPLC	146	Microwave ablation plus Camrelizumab	RFS, RFR for remaining lesions	Recruiting	Chang Chen, Dr Shanghai Pulmonary Hospital, Shanghai, China
NCT04026841		Interventional	II	Early MPLC with GGD	36	Sintilimab	ORR	Unknown	Jianxing He. The First Affiliated Hospital of Guangzhou Medical University
NCT04326751		Observational	NA	MPLC	20	NA	Tumor heterogeneity and Microenvironment	Not yet recruiting	Jun Wang, M.D. Peking University People’s Hospital Thoracic Surgery Department
NCT04982900	TERMGGO	Interventional	II	Multiple Synchronous GGOs	138	Furmonertinib, Placebo	Response rate	Recruiting	Hecheng Li, PhD, MD Ruijin Hospital Affiliated to Shanghai Jiao Tong University School of Medicine
NCT02833467		Observational	NA	MPLC	45	NA	Detection rate of cancer related genes	Unknown	Jun Wang, M.D. Peking University People’s Hospital
NCT03679936		Observational	NA	MPLC, IPM	120	PET/CT dynamic scan	Overall diagnostic sensitivity and specificity and ROC	Recruiting	Hongjun Jin, PhD. Fifth Affiliated Hospital, Sun Yat-Sen University
NCT04730453		Observational	NA	MPLC	30	ENB-guided ablation therapy plus VATS	ORR	Recruiting	Jiayuan Sun, MD, PhD. Shanghai Chest Hospital
NCT05662553		Interventional	NA	synchronous bilateral MPLC	172	ENB guided MWA combined with VATS, sequential surgery	Total perioperative complication rates	Recruiting	Hecheng LI, PhD, MD. Ruijin Hospital Affiliated to Shanghai Jiao Tong University School of Medicine

NA, not available. VATS, Video-assisted thoracoscopic surgery. SABR, Stereotactic Ablation Radiotherapy. MPLC, Multiple Primary Lung Cancer. sMPLC, Synchronous Multiple Primary Lung Cancer. RFS, Recurrence-Free Survival. RFR, Regression-free rate. ORR, Objective Response Rate. GGD, Ground Glass Density. GGOs, Ground-glass Opacities. IPM, Intrapulmonary metastases. ROC, Receiver operating characteristic. ENB, electromagnetic navigation bronchoscopy.

There was also a trial focused on the targeted therapy of multiple GGOs. Li et al. from Ruijin Hospital, affiliated to Shanghai Jiao Tong University School of Medicine, started a phase II clinical trial applying Furmonertinib (EGFR-TKI) to multiple synchronous ground-glass opacities in October 2021. Immunotherapy was also in GGOs clinical trials. He et al. from the First Affiliated Hospital of Guangzhou Medical University are recruiting GGOs MPLC patients for Sintilimab (anti-PD-1therapy).

Notably, all nine clinical trials are launched by Chinese researchers. Chinese researchers have paid much attention to MPLC in the past five years, and the results of the most established trials will come out within the next five years. We hope their findings can offer constructive suggestions to the management of MPLC, though, among the current trials, the maximal number of recruiting patients is 172, which might not be enough for a comprehensive illustration of this disease in our recognition.

Overall, the attention MPLC received from clinical trials does not match the significance of this disease in lung cancer, considering its high incidence. We are especially looking forward to more trials exploring the efficacy of immunotherapy in MPLC since the introduction of checkpoint inhibitors opens new avenues for cancer therapy. In addition, we look forward to the trials conducted on Caucasians, which might provide a valuable understanding of MPLC.

## Genomic updates about MPLC

Genomic characteristics of multifocal lung cancer started over 25 years ago (64). With the development of science and technology and the progress of gene research methods, several molecular methods such as DNA microsatellite analysis, array comparative genomic hybridization (aCGH), targeted sequencing, next-generation sequencing (NGS), and single-cell sequencing have been applied to profiling the characteristics of MPLC. We summarized the representative research on MPLC’s genomic profiling in recent years, as shown in **Table 4**.

**Table 4:** Representative genomic and molecular studies on MPLC within the recent 10 years.

Year	Reference	Materials	Genomic biomarker (methods)	Main Conclusions
2016	Schneider et al. PMID:27080983	60 patients with MPLCs	KRAS, EGFR, BRAF, PIK3CA, ALK, MET, ROS1, PIK3CA and p16 (mutations)	Concordance between histological and molecular staging was observed in 89% of adenocarcinomas and 56% of squamous cell carcinomas, and that the comprehensive genotypic and morphological assessment of surgically treated multifocal lung cancers is not sufficient to establish their clonal relationship and prognosis.
2016	Liu et al. PMID:27767028	6 patients with multiple synchronous lung cancers	WGS, WES and aCGH	Different lung cancers in the same individual may have distinct genomic profiles and can be driven by distinct molecular events.
2016	Yang et al. PMID:27796337	129 patients with MPLCs	EGFR, BRAF, ROS1 and KRAS mutations and EML4-ALK rearrangement	More than half of second primary lung cancers result from different mechanisms compared with primary cancers.
2017	Patel et al. PMID:28866070	11 patients with MPLCs and 8 patients with primary tumors and their metastasis	50 gene AmpliSeq Cancer Hotspot Panel v2	High mutational concordance was found in primary-metastatic pairs, and 8 of 11 MPLC patients had completely discordant mutations.
2017	Asmar et al. PMID:28647671	45 patients with primary tumors and their metastases and 69 patients with MPLCs	EGFR, KRAS, ALK and BRAF mutations	Oncogenic mutation concordance rate was 96% in patients with primary tumors and their metastase and 36% of MPLCs wrer indentified as primary by genomic profiling.
2017	Ma et al. PMID:29018192	4 patients with multiple synchronous lung cancers	WES	Each multicentric primary tumor harbors distinct oncogenic alterations, and robust evolutionary pressures can shape the expansion and constraint of genomic diversity simultaneously.
2017	Ma et al. PMID:29018192	5 patients with 18samples in the lung	Whole-exome sequencing and Clonality analysis	The results highlight the robust evolutionary pressures that simultaneously shape the expansion and constraint of genomic diversity, a principle that holds important implications for understanding tumor evolution and optimizing therapeutic strategies.
2018	Takahashi et al. PMID:30216592	37 multiple lung cancer patients	20 lung cancer‐related oncogenes mutations	Among 17 histopathological multiple priamry cases,a discordant of 47% (8 cases) was yielded by mutational evaluation. Multiplex mutational analysis could be a useful complementary tool for distinguishing between MP and IM in addition to histopathological evaluation.
2018	Roepman et al. PMID:29625247	50 patients with multiple lung lesions	50-gene panel and p53 protein expression	In 39% (19 cases) matching tumor samples, sequencing results were in contradiction to the initial immuno-histopathology diagnosis, and for about one-third of the patients, panel sequencing provided additional information to improve the differentiationbetween multiple primary lung cancers or pulmonary metastases.
2018	Chen et al. PMID:29092754	96 patients with MPLCs	EGFR, Tp53, KRAS, PIK3CA, and BRAF (somtatic mutation), and EML4-ALK, ROS1, RET (fusion gene)	A high rate of discordance of genetic alterations (89.7%) was found between cancers within individual patients.
2018	Santamaria et al. PMID:30032819	1 patient with three lung tumors	A targeted 50-gene DNA sequencing panel	Targeted DNA sequencing significantly increases diagnostic accuracy in patients with multiple lung tumors. NGS panels should be available for patients presenting with multiple lung tumors.
2018	Haratake et al. PMID:29254651	59 patients with multifocal lung cancer	PD-L1 expression	Among 43 patients with MPLC, disagreement of PD-L1 expression was i27.9% (12 patietns), and among 16 patients with pulmonary metastasis, disagreement of PD-L1 expression was 6.3% (1 patient). Higher levels of agreement of PD-L1 expression in pulmonary metastasis compared with in MPLC was found.
2019	Chang et al. PMID:31471310	60 patients with multifocal lung cancers	341-468 gene MSK-IMPACT NGS assay	Prospective histologic prediction was discordant with NGS in 17 cases (22%), particularly in the prediction of IPMs (44% discordant). Comprehensive NGS allows unambiguous delineation of clonal relationship among NSCLCs.
2019	Murphy et al. PMID:31103780	37 cases of multiple lung cancers	Mate-pair sequencing, EGFR, BRAF, KRAS, HRAS, NRAS, ALK, ERBB2, and MET (mutations) and ALK, ROS1, RET, and NTRK1 (gene fusions).	Histologic review appeared to misclassify lineage in 27% same-histology tumor pair comparisons, the highly unique nature and prevalence of chromosomal rearrangement in lung cancers provide a useful and definitive technique for calling lineage in multifocal lung cancer.
2020	Li et al. PMID:31699841	154 subsolid nodules samples from 120 patients	Whole-exome sequencing	Mutations in EGFR were the most prominent and significant variation, followed by those in RBM10, TP53, STK11 and KRAS.
2020	Higuchi et al. PMID:32093372	37 patients with multiple lung cancers	A panel covering the exons of 53 lung cancer-related genes	In multicentric primary lung cancers, the driver mutation profile was mutually exclusive among the individual tumors, while it was consistent between metastasized tumors and the primary lesion.
2021	Hu et al. PMID:34887263	112 patients with 255 tumors	1021-gene panel	MPLCs are driven by different molecular events and often exhibit low TMB, low PD-L1, and a heterogeneous immune infiltration landscape. The most frequently mutated genes were EGFR (56%), ERBB2 (12%), TP53 (12%), and BRAF (11%). 87 (77.7%) patients were with diverse genomic profiles, and 61 (54.5%) shared at least one putative driver gene between different tumors presented more aggressive tumors.
2021	Motohiro et al. PMID:33707471	17 patients with 38 specimens	409 cancer-associated genes panel	Comprehensive genetic analyses suggested different mutation profiles in tumours within the same individuals, with some exceptions. EGFR, KRAS, TP53, or PARP1 mutations were concomitantly detected in some MPLC cases.
2021	Daryn et al. PMID:33845213	40 patients with multiple lung cancers	A gene panel (Ion 318 Chip v2 or Ion 314 Chip) greater than 50 genes, including ALK, BRAF, EGFR, FGFR1, KIT, KRAS, MET, NRAS, PIK3CA, PTEN, RET, and TP53.	Mutational profiling was concordant with clinicopathologic diagnosis in most cases, and seven cases (17.5%) revealed shared mutations suggesting metastatic disease and this was associated with a substantial reduction in overall survival.
2021	Zhang et al. PMID:33820821	1 patient received immunotherapy	WES, IHC, single-cell sequencing	The genomic disparities among responding and non-responding nodules were detected at various levels, suggesting that neoadjuvant PD-1/PD-L1 inhibitors alone may not be optimal for MPLC.
2022	He et al. PDIM:35874770	3 GGOs patients	Single-cell sequencing	Cancer cells in the S components, which showed relatively malignant phenotypes, were likely to originate from both the GG and S components and monitor the surrounding tumor microenvironment (TME) through an intricate cell interaction network.
2022	Yu et al. PMID:36029220	334 resected pulmonary nodules from 262 Chinese patients	A custom 1021-gene panel, IHC, imaging data	They demonstrates a driver landscape of radiologically detectable GGO-associated pulmonary nodules in Chinese patients and supports that different driver patterns in RTK/RAS pathway are corresponding to different radiologic features.
2022	Wang et al. PMID:36531058	141 and 44 lesions from single and multiple primary lung adenocarcinoma	Next-generation sequencing-based YuanSu450TM gene panel	Mutation analysis of SP- and MP-LUAD patients could identified genomic alterations and evolutionary trajectories underlying MP-LUAD and will provide new insights into the oncogenesis of MP-LUAD and useful information for development novel approach to target MP-LUAD.

Before the widespread of NGS, gene panels containing a few oncogenic/tumor-suppressor genes (usually 1 to 5 genes) and chromosome alterations in MPLC were the focus, which was far from enough for profiling the MPLC genome. Using target sequencing, many researchers tested genes such as EGFR, KRAS, and p53 in MPLC (65–67). Chromosome alterations such as copy number analysis (27, 68), microsatellite instability (MSI) (69), loss of heterozygosity (LOH) analysis (69, 70), and X chromosome inactivation (37) were also detected in MPLC by genomic hybridization (aCGH). Those previous researchers tried to find out the difference and similarities of lesions of MPLC (37, 40), but inefficient technology hinders our in-depth understanding of the MPLC genome.

In the era of NGS, various sequencing approaches and analysis methods have emerged, fueling a wave of genomic research on MPLC.

*1. The distinction between MPLC and IPM*

The precise differentiation between MPLC and IPM is one of the driving forces of the genomic exploration of MPLC. Genomic profiling information challenged the traditional clinicopathologic criteria of MPLC. In the era of next-generation sequencing (NGS), the application of large gene sequencing panels, whole-genome sequencing (WGS), and chromosome rearrangements helped us distinguish MPLC from IPM

In 2018, Santamaria et al. (71) conducted a targeted 50-gene DNA sequencing panel on a lung cancer patient with three lesions. Their results supported that all three lesions were independent in mutation background, indicating they were MPLC rather than advanced lung cancer. In 2019, similar research was conducted by Chang et al. using an NGS assay covering up to 468 cancer-related genes in 60 patients, showing that the histologic classification was contrary to the judgment based on NGS in 22% of all cases, especially in the prediction of IPMs, in which the misdiagnosis rate was up to 44% (72).

Whole-genome sequencing (WGS) and whole-exome sequencing (WES) are more comprehensive in profiling genomic features than targeted sequencing. In 2016, Liu et al. (73) were the first to portray the genomic architecture of MPLC using WGS and WES. They collected 16 tumor samples from six patients. One patient was identified as having metastasis under histopathological standards and was classified as primary based on genomic sequencing results. In 2018, Li et al. (74) performed exome sequencing on two non-smoker patients with multiple GGOs (patient 1 had seven subsolid nodules and one pure GGO, and all the six nodules of patient 2 were pure GGOs). Results showed that two subsolid nodules in patient one and two pure GGOs in patient 2 were clonally-related, respectively, indicating that intrapulmonary metastasis could occur among GGOs, even in pure GGOs, which reshaped people’s impression of multiple GGOs.

Recently, chromosome rearrangements were reported to be an effective tool for this discrimination (29, 38). In 2014 and 2019, Murphy et al. applied mate-pair sequencing (MPS) to 11 and 37 patients with multifocal lung cancer with known metastasis lesions, respectively. In 2019, a lung cancer NGS panel was applied to 17 of 37 patients. Both the two studies of Murphy et al. showed that DNA rearrangements generated by MPS performed well in the lineage calling of MPLC.

*2. Multi-omics analysis of MPLC*

Nowadays, multi-omics analysis of MPLC is used to confirm the heterogeneity and discuss the tumor immune microenvironment of multiple nodules in MPLC. Clonal evolution is a method to portray the evolution of MPLC. PyClone is a statistical model introduced by Roth et al. (75) for inference of clonal population structures in cancers. In 2017, Ma et al. (76) sequenced four patients with multiple synchronous lesions by WES and performed phylogenetic analysis showing clonal architectures. Despite the identical genetic background and environmental exposure, they found significant genomic heterogeneity in individual patients’ inter-focal and intra-focal levels. Currently, this method has not been well performed in MPLC.

In 2020, Wang and his team (77) first integrated the radiological image data of mGGOs into their genomic analysis to investigate the intratumoral heterogeneity and clonal relationship of multifocal GGOs. They found that the differences between subsolid nodules (SSNs) and advanced-stage LUADs at a genomic level were unraveled. Although multicentric origin was predominant, they also detected early metastatic events among multifocal SSNs. Similarly, Yu et al. (78) performed a custom 1021-gene panel sequencing of 334 resected pulmonary nodules presenting as GGO from 262 Chinese patients. They compared gene pathways enriched in different GGOs (pure GGOs and mixed GGOs) through genomic profiling. They demonstrate a driver landscape of radiologically detectable GGO-associated pulmonary nodules in Chinese patients and support that different driver patterns in RTK/RAS pathway correspond to different radiologic features.

Single-cell sequencing technology has been a brilliant new technology for tumor analysis in recent years. It studied the changes in the multicellular microenvironment of the tumor by studying the cell composition in tumor tissue. In 2021, Zhang et al. (21) performed WES, Immunohistochemistry (IHC), single-cell RNA seq, TCR (T cell receptor repertoire)-seq in the multiple nodules of one MPLC case undergoing neoadjuvant pembrolizumab treatment. The genomic disparities among responding and non-responding nodules were detected at various levels, suggesting that neoadjuvant PD-1/PD-L1 inhibitors alone may not be optimal for MPLC.

In 2022, 10x Genomics single-cell RNA-seq was used in GGOs by Li et al. (79), and they identified transcriptomic differences in the vital signaling pathways of tumor and immune cells within GGOs, implying the transcriptomic heterogeneity of this disease. Similarly, He et al. (80) macro-dissected the solid (S) components and ground-glass (GG) components of mGGO and performed single-cell sequencing analyses of six paired components from three mGGO patients. They found that cancer cells and macrophages were the dominant cell types in the S and GG components, respectively. Cancer cells in the S components, which showed relatively malignant phenotypes, were likely to originate from both the GG and S components and monitor the surrounding tumor microenvironment (TME) through an intricate cell interaction network.

In summary, with the progress of science and technology, our perceptions about MPLC are growing. Thanks to the initial exploration of the heterogeneity of MPLC, to the later identification of MPLC and IPM, and finally to the use of advanced sequencing technology to clarify the development mechanisms/tumor immune microenvironment, our understanding of MPLC is now deeper and it is expected to increase in the future.

## Summary

From clinical management to genomic profiling, this review gives a comprehensive description of MPLC, which is a highly focused malignancy by clinicians. We hope our work can enlighten more profound reflections about MPLC from the public.

## Author contributions

JH, SG, and CL designed this study and provided funding support. HT, GB, and ZY drafted the manuscript and completed the tables. PC, JX, TL, TF, BW, and CX collected the references and completed the tables. All authors contributed to the article and approved the submitted version.
